# Impact of the Amphoteric Nature of a Chelating Surfactant on its Interaction with an Anionic Surfactant: A Surface Tension and Neutron Reflectivity Study of Binary Mixed Solutions

**DOI:** 10.1021/acsomega.3c07547

**Published:** 2024-02-26

**Authors:** Ida Svanedal, Håkan Edlund, Magnus Norgren, Sushil K. Satija, Adrian R. Rennie

**Affiliations:** † Surface and Colloid Engineering, FSCN Research Centre, Mid Sweden University, Sundsvall SE-851 70, Sweden; ‡ NIST Center for Neutron Research, 100 Bureau Drive, Gaithersburg, Maryland 6100, United States; § Macromolecular Chemistry and Centre for Neutron Scattering, Uppsala University, Ångström Laboratory, Box 538, Uppsala SE-75121, Sweden

## Abstract

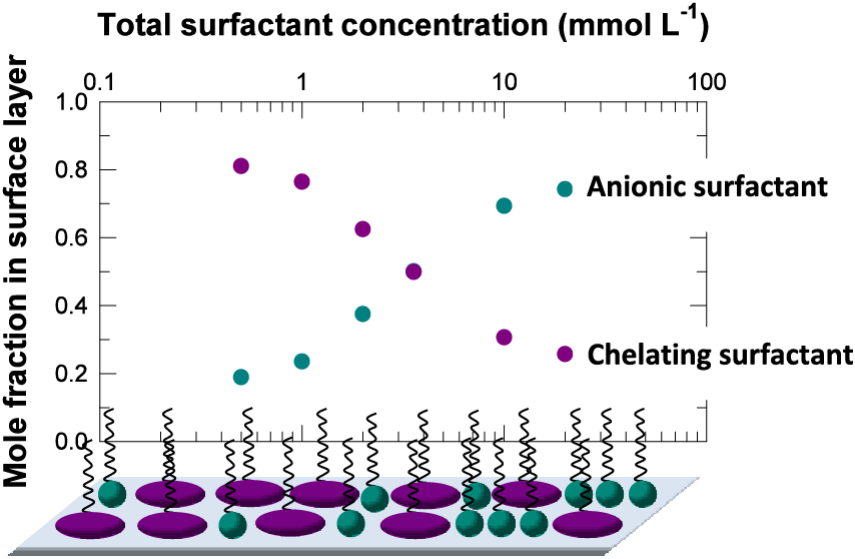

2-Dodecyldiethylenetriaminepentaacetic acid (C_12_-DTPA) is a chelating, amphoteric surfactant with a bulky headgroup containing eight pH-responsive groups. The hypothesis was that the amphoteric nature of the chelating surfactant would affect the interaction with another surfactant and, consequently, also the composition of mixed surface layers. Binary mixed monolayers of C_12_-DTPA and the anionic surfactant sodium dodecyl sulfate (SDS) were examined using neutron reflection and surface tension measurements. The experiments were conducted at pH 5, where the C_12_-DTPA monomers carried a net negative charge. Surface excess calculations at low total surfactant concentration revealed that the chelating surfactant dominated the surface composition. However, as the concentration was raised, the surface composition shifted toward an SDS-dominant state. This phenomenon was attributed to the increased ionic strength at increased concentrations, which altered the balance between competing entropic forces in the system. Interaction parameters for mixed monolayer formation were calculated, following a framework based on regular solution theory. In accordance with the hypothesis, the chelating surfactant’s ability to modulate its charge and mitigate repulsive interactions in the surface layer resulted in favorable interactions between the anionic SDS and negatively charged C_12_-DTPA monomers. These interactions were found to be concentration-dependent, which was consistent with the observed shift in the surface layer composition.

## Introduction

**Figure 1:**
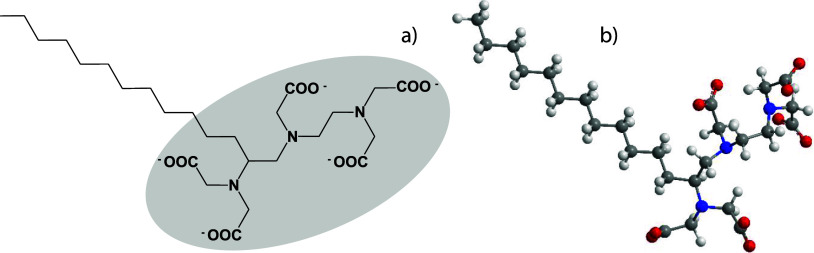
(a) Structure of the chelating surfactant C_12_-DTPA at high pH, where all eight donor atoms are deprotonated. (b) Ball and stick model of C_12_-DTPA at high pH.

A chelating surfactant is a surface-active polydentate ligand that forms strong coordination complexes with metal ions. Metal ion coordination and conditional stability constants of 2-dodecyldiethylenetriaminepentaacetic acid (C_12_-DTPA), the chelating surfactant examined in this study, have been reported previously.^1^ Since the donor atoms are also pH-responsive, this results in an amphoteric surfactant, with the potential for changes in charge and charge distribution that depend on the prevailing pH. C_12_-DTPA contains eight pH-responsive groups in its hydrophilic part, three tertiary amine and five carboxylate groups, and the structure consequently involves eight p*K*_*a*_-values. The dissociation behavior of C_12_-DTPA has been investigated and compared with that of the conventional chelating agent diethylenetriaminepentaacetic acid (DTPA) in a previous study,^2^ although determination of the exact p*K*_*a*_-values of C_12_-DTPA has not yet been the focus of our investigations. At the lowest pH, the headgroup carries a positive charge of +3 when all eight donor atoms are protonated. As the pH increases, the molecule transforms into several zwitterionic species, containing both positive charges from protonated nitrogen atoms and negative charges from deprotonated carboxyl groups. Ultimately, a negative charge of −5 is reached at the highest pH when all eight donor atoms are deprotonated. A difference of less than four between successive p*K*_*a*_-values implies that there is overlap between the pH range of different species in equilibrium, and the closer they are the larger the overlap. Apart from the extreme pH levels, a diverse array of surfactant species with various charges is present due to the overlapping p*K*_*a*_-values of the functional groups. Consequently, instead of a singular surfactant species, a mixture of differently charged species coexists at intermediate pH. Furthermore, it is reasonable to assume that the net charge may differ between free monomers in solution, surfactants in micelles, and surfactants in surface layers. Monomers in solution will be more ionized and surface molecules less ionized because of intermolecular repulsion between adjacent charged surfactant molecules packed closely together. In the context of the current study, which utilizes a pH of 5 for all experiments, the predominant species is zwitterionic but has a net negative charge. Because of the complex dissociation behavior, the structure of C_12_-DTPA is shown in Figure 1 at high pH.

We have reported the surface tension of aqueous solutions of the chelating surfactant C_12_-DTPA in previous studies and shown that the data are consistent for the normal hydrogenous material and for surfactant synthesized with deuterated hydrocarbon tails.^2,3^ Rather than observing the typical abrupt transition from decreasing surface tension to a relatively stable plateau in the plot depicting surface tension against increasing concentration, we found consistently a gradually flattened curve that reaches a minimum. This was followed by an increase in surface tension from around the critical micelle concentration (cmc), determined to 20 ± 3 mmol L^–1^ at pH 5 by using NMR diffusion measurements.^2^ It should be noted that the minimum in the surface tension is reproducible, even for different samples that have been thoroughly purified.

Our previous studies utilizing neutron reflection to analyze the surface excess and thickness of adsorbed layers of C_12_-DTPA at the air/water interface have established correlations with the surface tension data. These correlations serve to confirm that the quantity of material present at the surface diminishes as the concentration increases, particularly once micelles form within the bulk solution. Consequently, this suggests that there is a discernible alteration in the solution’s activity beyond the cmc.^3^

The thermodynamics of interfacial layers is usually interpreted in a first simple way according to the model of Gibbs that relates the gradient in plots of surface tension against the logarithm of the concentration or activity to the surface excess. This approach becomes slightly more complicated for ionic surfactants, as allowance for the dissociation of the adsorbate must be made in the evaluation of the surface excess. Some simplifications are possible when there is a large excess of electrolytes. There are more extensive treatments for some mixtures of surface-active molecules. The Butler equations were derived on the basis of ideal mixing of the components.^4^ A widely used approach based on the ideas of Clint^5^ and Rubingh and Holland^6^ has incorporated a binary interaction parameter, β, that allows for a change of the free energy in a mixed layer beyond that from a simple entropy of mixing. This is often interpreted as a change of internal energy and as such would represent a “regular solution” model for the mixing of molecules in the surface layer. Penfold and Thomas have provided two helpful recent reviews that describe a number of further complexities.^7,8^ In general, the activity of a solution may not be entirely constant at concentrations above the critical micelle concentration, as micellar organization can change. For mixed systems, interaction parameters may be different in adsorbed monolayers and when determined for the assembly as micelles because of the different packing arrangements and degrees of dissociation. Zwitterionic surfactants, polyvalent amphiphiles, and materials that are sensitive to pH can show significant further effects. In many cases, mixtures with these materials are considered to first approximation as depending on a single parameter that describes pairwise interactions. Without detailed information about the structure and arrangement of the components or data for different temperatures, it is not possible to assess how much difference is due to internal energy and how much is due to entropy of disorder or composition. Interaction parameters, which might be semiempirical, for mixed monolayer formation can be calculated from surface tension, using the approach described by Holland and Rubingh.^6^

Previous studies have provided evidence that amphoteric surfactants engage in interactions with ionic surfactants through the acceptance or donation of protons to the aqueous solution. This process enables them to modify their electric charge and enhance the strength of their interaction with adjacent surfactant molecules.^9−12^ By adaptation of the protonation of functional groups to minimize electrostatic repulsions, the surfactant interactions become more favorable due to reduced counterion binding. In turn, the changes of interaction and composition in mixed layers and micelles thereby possibly alter the entropy. This effect is less pronounced if background electrolytes are added. In the present study, monolayers at the air/water interface of binary mixtures of the chelating surfactant C_12_-DTPA and the anionic surfactant sodium dodecyl sulfate (SDS) are investigated, without added electrolytes, by using neutron reflection and surface tension measurements. The purpose is to examine the correlation between the composition in the monolayer and the interactions between the two surfactants, and to evaluate the impact of the chelating surfactant’s amphoteric nature on those interactions. This is done by evaluating surface excess concentrations and vertical extensions of the two surfactants at different concentrations and mixing ratios, calculating interaction parameters between the surfactants, and comparing the results regarding the surface composition obtained from the two different techniques. In the derivation of equations for determining interaction parameters, electrical effects are ignored. For simplicity, it is recommended to maintain a constant ionic strength by adding a swamping electrolyte when studying ionic surfactants. By following this approach, interaction parameters that correctly describe the system over all mixing ratios should be obtained.^13^ It is, therefore, crucial to acknowledge that in systems such as the one described here, where the mixture composition results in varying ionic strength, the interaction parameters may also exhibit variations with respect to the composition. In previous papers on the subject, we have also discussed reasonable limitations in order to treat a mixed system of C_12_-DTPA and another surfactant as a binary mixture.^11,12^ The following limitations and assumptions are assumed: (i) The pure C_12_-DTPA is defined based on the distribution of differently charged species at a specific pH, and this would include possible rearrangements of the hydrogen atoms within the headgroup. (ii) The presence of the second surfactant can influence the dissociation of C_12_-DTPA surfactants in the mixed monolayers, potentially resulting in a distribution of C_12_-DTPA species that differs from the distribution observed in the pure C_12_-DTPA layers. (iii) The interaction parameter is predominantly influenced by the strongest interactions within the systems, and as such, the calculated interaction parameter primarily describes these specific interactions.

## Experimental Section

### Materials

2-Dodecyldiethylenetriaminepentaacetic acid (C_26_H_47_N_3_O_10_), solid powder, was delivered by Syntagon AB. The synthesis and analyses have been reported previously.^2^ The deuterated analogue, 2-dodecyldiethylenetriamine-*d*_*25*_ pentaacetic acid, (C_26_D_25_H_22_N_3_O_10_) was prepared at Mid Sweden University.^3^

Sodium dodecyl sulfate (NaC_12_H_25_SO_4_, 99%, Sigma-Aldrich), deuterated sodium dodecyl sulfate (NaC_12_D_25_SO_4_, 98%, Cambridge Isotope Laboratories, Inc.), sodium hydroxide (NaOH, 99,2%, VWR chemicals), and deuterium oxide (D_2_O, 99.9%, Cambridge Isotope Laboratories, Inc.) were used as received. Water used was Milli-Q grade.

The elemental compositions of the surfactants are known, and so the neutron scattering length for each can be calculated from known values of the cross-section for the elements.^14^ The relevant values are given in Table 1.

**Table 1:** Neutron Scattering Lengths for Surfactants

material	chemical formula	*b*_m_ (fm)
sodium dodecyl sulfate, SDS	NaC_12_H_25_SO_4_	15.93
deuterated SDS	NaC_12_D_25_SO_4_	276.22
C_12_H_25_-DTPA	C_26_H_47_N_3_O_10_	127.99
C_12_D_25_-DTPA	C_26_D_25_H_22_N_3_O_10_	388.28

### Sample Preparation

For neutron reflectometry measurements, samples were prepared in null reflecting water (water that contains 8% by volume of D_2_O), from either one deuterated surfactant (pure surfactant) or one deuterated surfactant and the other hydrogenous surfactant (binary mixtures). If necessary, the pH was adjusted to 5 by addition of sodium hydroxide. Two sets of each mixture were prepared: one containing deuterated C_12_-DTPA and hydrogenous SDS and the other one containing hydrogenous C_12_-DTPA and deuterated SDS. This was done to enable quantification of both surfactants separately in the mixtures.

For surface tension measurements, samples were prepared from hydrogenous surfactants dissolved in water. If necessary, the pH was adjusted to 5 by addition of sodium hydroxide.

### Neutron Reflection

Neutron reflectometry measurements were performed on the NG7 reflectometer at the NIST Center for Neutron Research.^15^ Data were recorded with a linear detector using an incident wavelength, λ, of 4.768 Å. The grazing angle of incidence, θ, and the position of the detector were scanned to provide a range of momentum transfer, *Q* (=(4π/λ) sin θ), from 0.01 to 0.15 Å^–1^. Samples of the solutions were placed in polytetrafluoroethylene (PTFE) troughs that were placed on an active antivibration table on the sample translation stage. Covers with thin aluminum foil windows were used to reduce the evaporation and exchange of water with the air. The direct beam was measured on a linear detector by moving the sample out of the beam. The data reduction involved integration of the intensity of the peak for the specular reflection on the position sensitive detector and subtraction of background scattering observed in adjacent regions as well as normalization to the direct beam intensity.

### Modeling Neutron Reflection Data

Water that contains 8% by volume of D_2_O will have an average neutron scattering length density of zero, and so the interface between air and this composition of water would, on its own, not give rise to any specular reflection. This is often described as null reflecting water, NRW. A solution with a surface excess of material that has a different scattering length density to zero can give rise to simple determination of the surface excess in terms of the scattering length. For a solution with a mixture of surface-active species, it is necessary to use multiple contrasts, for example, hydrogen and deuterium labels of the various different surfactant molecules so that the excess for the individual components can be determined.^16^

In the case of thin adsorbed films, the measurements of reflectivity at small *Q* are usually adequately modeled as a single uniform layer that is conveniently characterized by an average scattering length per unit area (*b*_A_ = *b*/*a*) and a thickness, *t*. Although many authors describe the layer in terms of a scattering length density, ρ, that would be equal to *b*/*at,* this is less useful for fit procedures as ρ and *t* tend to be strongly inversely correlated whereas for thin layers, *b*_A_ and *t* will be independent variables.^17^

For a single component, m, the surface excess Γ_m_ in molecules per unit area would simply be calculated as *b*_A_/*b*_m_, where *b*_m_ is the total scattering length for the molecule m. In some cases, it is possible to prepare mixtures such that one component has a large scattering length, and the others are approximately zero and such systems allow easy determination of the surface excess of individual components. When this approximation is not adequate, it is quite straightforward to make fits to combinations of data sets with different contrasts that allow for the simultaneous presence of two or more components with significant contrast. This will still involve modeling for an individual data set as a single layer but in this case, the scattering length per unit area is given by
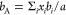
1where *x*_*i*_ is the molar fraction of component *i* with molecular scattering length *b*_*i*_ in the surface layer and the sum is taken over all the components. At least as many independent data sets with different contrasts will be needed as the number of components. Further data sets can improve the modeling and indicate whether the assumptions about thin uniform layers are reasonable. In practice, it is convenient to use software that is designed specifically to fit the surface excess and thickness of multiple components such as mcatamaran.^18^ This procedure is required for the mixture of SDS and C_12_DTPA as it is clear from the values in Table 1 that the scattering length for the hydrogenous chelating surfactant is almost 50% of the value for the deuterated SDS and so can contribute significantly to the reflectivity in that mixture.

### Surface Tension Measurements

The surface tension was measured with a Krüss K6 tensiometer and a platinum/iridium du Noüy ring at a temperature of 22 °C. Each sample was measured five times consecutively, and the mean values are reported.

## Results and Discussion

The surfactant monolayers at the air/water interface of the binary surfactant mixtures were studied using neutron reflectometry followed by surface tension measurements on the same solutions to evaluate the composition in the monolayer and interaction parameters between the two surfactants.

### The Distribution of Surfactants in Mixed Monolayers—Effects of Total Surfactant Concentration

**Figure 2:**
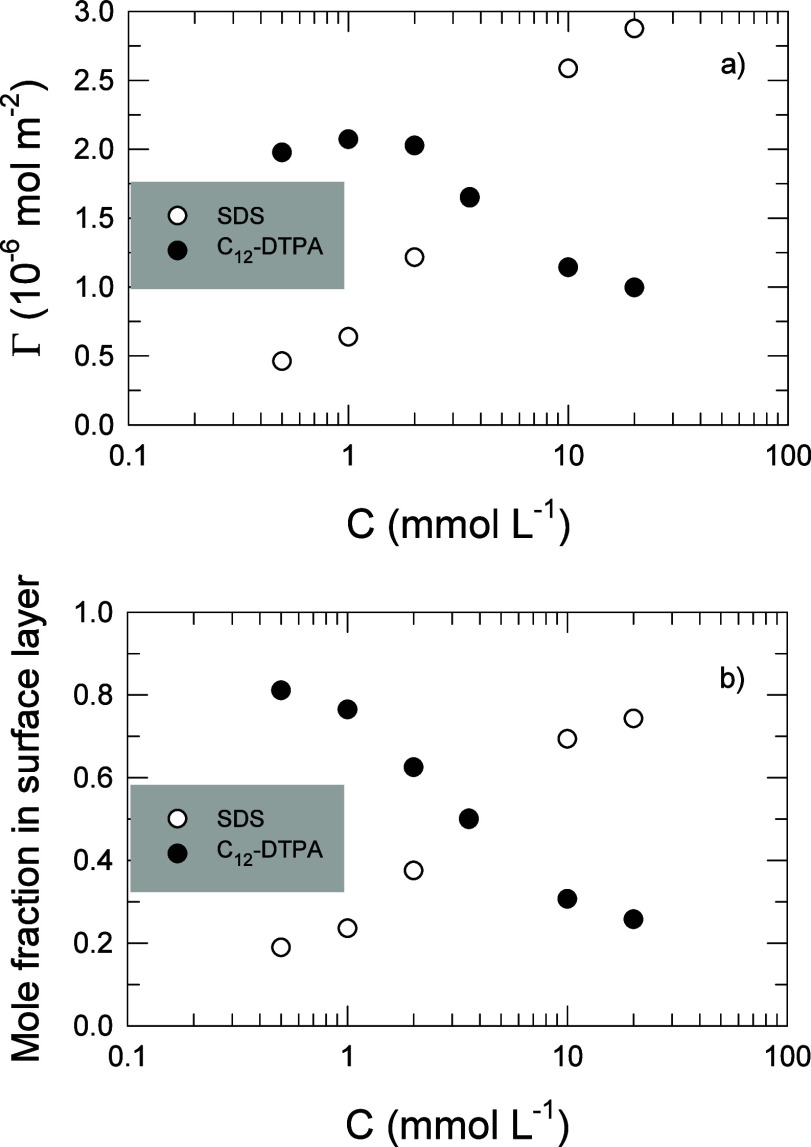
(a) Surface excess (Γ) of C_12_-DTPA and SDS in equimolar solutions as a function of total surfactant concentration, *C*. (b) Mole fraction of C_12_-DTPA and SDS in the surface layer as a function of total surfactant concentration, *C*. Values are calculated from the fits to neutron reflectivity data. Typical uncertainties for fits of the surface excess are 3%. (As elsewhere in this article, the uncertainties and derived errors are reported as one standard deviation.)

Equimolar mixtures of C_12_-DTPA and SDS were studied for a range of different total surfactant concentrations using neutron reflection measurements that are shown in Figure S1, and the surface excess of the respective surfactant in mixed monolayers at the air/water interface was calculated from the model fits to these measurements (the parameters for the fits are listed in Table S1). The surface excess concentration (Γ) is defined as the difference between the interfacial concentration of a substance and the concentration at a virtual interface in the interior of the bulk phase. Surface excess and mole fraction of the individual surfactants, as a function of the total surfactant concentration, are shown in Figure 2a,b, respectively.

We start the analysis of Figure 2 by looking at the low concentration, where the chelating surfactant dominates the surface adsorption. Adsorption of surfactants to a surface is driven by the hydrophobic effect, expelling a polar molecule from the interior of the water. For electrostatic reasons, adsorption of ionic surfactants is accompanied by a certain degree of counterion binding. The counterion binding will, however, counteract the adsorption since it reduces the entropy of the counterions, referred to as an entropy penalty of counterion binding.^19^ As stated in the Introduction, the chelating surfactant has a negative net charge at the investigated pH of 5, but its amphoteric character enables the structure to reduce its negative charge through increased protonation when adsorbed at the surface, resulting in reduced counterion binding to the surface.^19^ SDS, on the contrary, is not able to adapt its charge by protonation and is less sensitive to the pH. Since the adsorption is limited by the loss in counterion entropy, this is likely the reason why C_12_-DTPA is predominantly adsorbed over SDS at low concentrations. The entropy of mixing will, however, favor simultaneous adsorption of the two surfactants, resulting in a mixed monolayer, as seen from the presence of both surfactants throughout the whole concentration range examined.

Increasing the solution concentration gradually increases the surface excess of SDS, at the expense of C_12_-DTPA. This results in an equimolar surface excess for the two components at a total surfactant concentration around 3.5 mmol L^–1^. The cmc of the equimolar mixture of the nondeuterated (hydrogenous) surfactants has been determined to be 6 ± 2 mmol L^–1^ in a previous study,^11^ and this implies that the equimolar surface excess occurs at a concentration slightly below the cmc for the mixture. At the highest concentrations investigated, SDS dominates the surface. The underlying cause of this exchange of surfactant type at the air/water interface is supposedly the increase in ionic strength at increasing concentrations. Electrolyte addition is known to decrease the penalty of counterion binding, thereby facilitating the adsorption of ionic surfactants.^19^ This will amplify the effect of the other main structural difference between the two surfactants, i.e., the size of the headgroups. As shown in Figure 1, C_12_-DTPA has a rather bulky headgroup, whereas SDS has a smaller headgroup. As the concentration, and subsequently the ionic strength, increases, the advantage that the amphoteric nature plays on the counterion binding becomes less pronounced, and the steric effects associated with the bulky headgroup of the chelating surfactant become significant instead. This will gradually favor the adsorption of the less bulky SDS, at increasing concentrations. Slight concentration-dependent variations in the surface composition have been reported previously for 50:50 mixtures of simple zwitterionic and anionic surfactants,^20,21^ although not as pronounced as the results reported here. Li et al. studied mixtures of SDS and the zwitterionic surfactant *N*-dodecyl-*N*,*N*-dimethyl-3-ammonio-1-propane sulfate (C_12_-sulphobetaine).^20^ In equimolar mixtures, they found that the adsorbed fraction of SDS increased slightly, from 0.32 to 0.41, when the total surfactant concentration increased from 0.22 to 6.6 mmol L^–1^. They reported the cmc of the 50:50 mixture to be 0.3 mmol L^–1^. Hines et al. studied the surface composition of SDS and n-dodecyl-*N*,*N*-dimethyl-aminoacetate (C_12_-betaine) mixtures and reported that the mole fraction of SDS at the surface increased from 0.30 to 0.36 when the surface pressure increased from 17 to 32 mN m^–1^.^21^ In the system studied in the present work, the change of total surfactant concentration from 0.5 to 20 mmol L^–1^ caused the mole fraction of SDS in the surface layer to increase significantly, from 0.19 to 0.74. Although the literature on the topic is quite sparse, comparison with previous studies involving mixtures of simple zwitterionic surfactants indicates that the amphoteric nature of C_12_ DTPA has an impact on the observed results.

**Figure 3:**
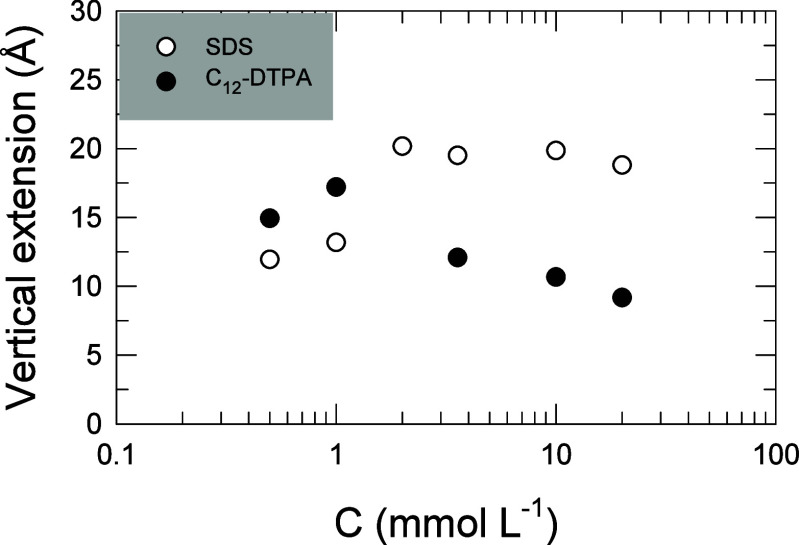
Vertical extension of C_12_-DTPA and SDS in equimolar solutions as a function of the total surfactant concentration. Values are calculated from the fits to neutron reflectivity data. Typical uncertainties for fits of the vertical extension are less than 0.1 Å, except for the lowest values of vertical extension where the errors are less than 0.2 Å.

Information regarding the thickness of the adsorbed layer at the air–water interface can also be derived from neutron reflection measurements. Here, the two surfactants were traced individually, and the result is, therefore, discussed in terms of vertical extension of the respective surfactant rather than thickness. As above, calculations were based on the experiments on equimolar surfactant solutions at increasing total surfactant concentrations, see Figure 3.

### The Distribution of Surfactants in Mixed Monolayers—Effects of Surfactant Composition

**Figure 4:**
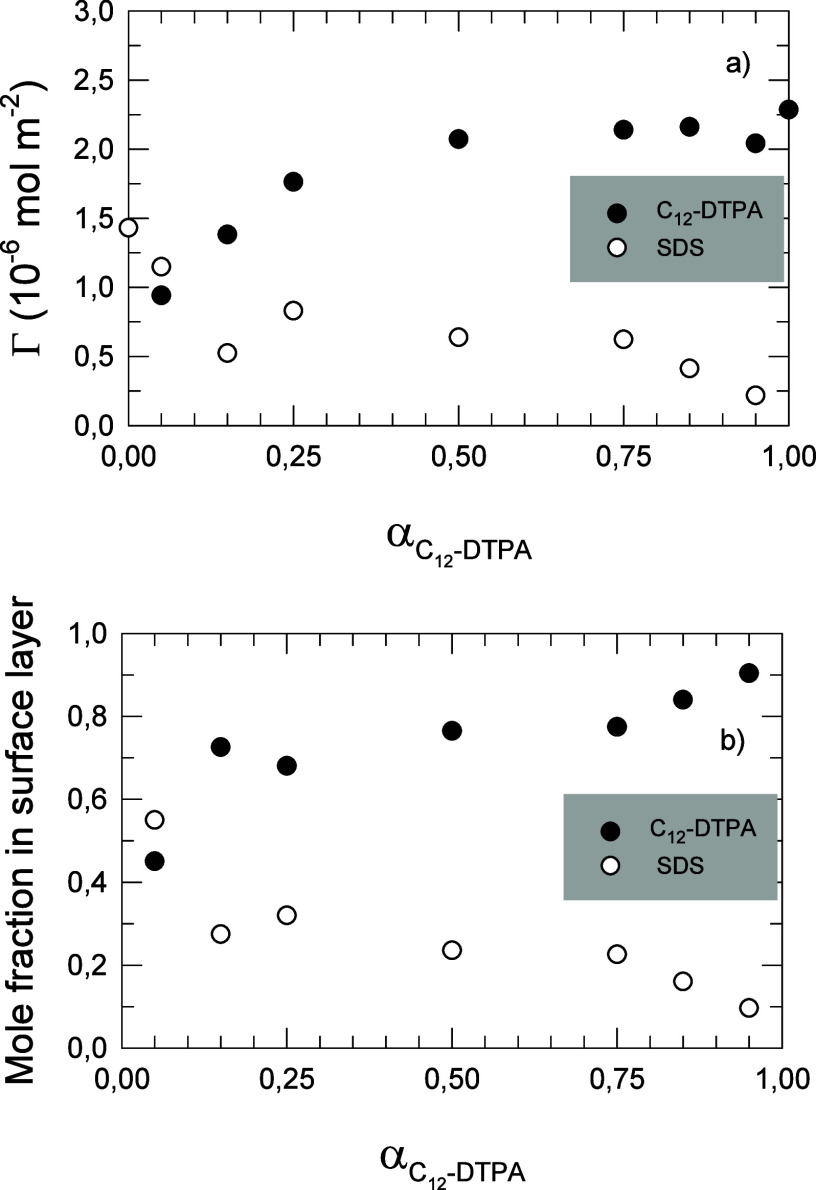
(a) Surface excess (Γ) for C_12_-DTPA and SDS as a function of mole fraction C_12_-DTPA in solution (α), at a fixed total surfactant concentration of 1 mmol L^–1^. (b) Mole fraction of C_12_-DTPA and SDS in the surface layer as a function of mole fraction C_12_-DTPA in solution (α), at a fixed total surfactant concentration of 1 mmol L^–1^. Values are calculated from the fits to neutron reflectivity data. Typical uncertainties for surface excess are 3%.

The distribution of the two surfactants in mixed monolayers was further evaluated at a low total surfactant concentration. Several different mixing ratios were examined, while keeping the total surfactant concentration at a fixed value of 1 mmol L^–1^. Figure 4a shows the surface excess of C_12_-DTPA and SDS as a function of the mole fraction of C_12_-DTPA in solution (α). The reflectivity data, model fits, and parameters are shown in Figure S2 and Table S2.

Note that throughout this text, mole fractions are given on a surfactant only basis. Comparing the pure surfactants in Figure 4a, C_12_-DTPA has a higher surface excess than SDS. This is also the case for most of the examined mixing ratios, indicating that C_12_-DTPA is more surface active than SDS at this concentration. Figure 4a can be compared with the low concentration range in Figure 2, and the results are consistent. The results in Figure 4a are most likely a consequence of the mechanism discussed earlier, i.e., that C_12_-DTPA is reducing its negative net charge and is thereby favorably adsorbed to the surface due to less counterion binding. The only mixing ratio for which SDS is present at a higher concentration at the surface compared to C_12_-DTPA is when the solution consists of 0.95 mole fraction of SDS, for all other mixtures C_12_-DTPA is dominating the surface excess.

Figure 4b shows the mole fraction of C_12_-DTPA and SDS at the air/water interface as a function of mole fraction of C_12_-DTPA in solution (α), at a total surfactant concentration of 1 mmol L^–1^. While the amount of surfactant adsorbed at the interface is determined by the balance between the hydrophobic effect driving the adsorption and the entropic and/or steric forces counteracting the adsorption, interactions between the surfactants in the surface layer cause the surface composition, i.e., the relative amount of the two surfactants at the surface, to deviate from the solution composition. The entropy penalty associated with counterion binding tends to favor the adsorption of the chelating surfactant, whereas the entropy of mixing in the surface layer favors the mixed adsorption of the two surfactants. The composition of the surface layer will be a result of the balance between these two effects. As seen in the figure, the C_12_-DTPA/SDS mixtures tend toward a C_12_-DTPA mole fraction of 0.75 in the mixed monolayer at the surface, at this specific concentration. As shown in Figure 2, the surface composition changes with the total concentration, resulting in increased SDS content in the surface layer at increased total surfactant concentration. As will be discussed later in the section Maximum synergism in the surface tension reduction efficiency—the optimum composition, even though the surface composition changes with concentration, this phenomenon of the surface composition tending toward a specific ratio between the surfactants over a large solution composition persists.

### Surface Tension Measurements and Synergism in Mixed Monolayers

**Figure 5:**
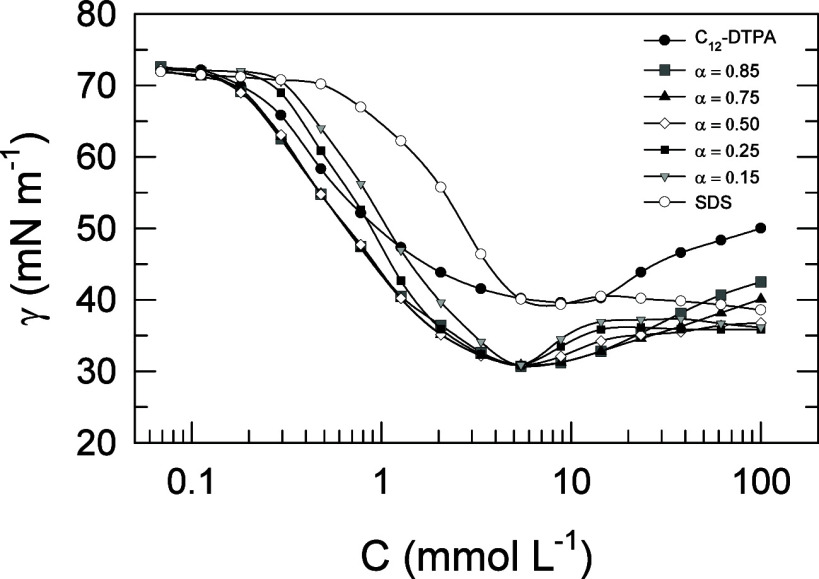
Surface tension as a function of total surfactant concentration for C_12_-DTPA, SDS, and the five mixtures at different mole fractions of C_12_-DTPA (α). The lines between data points are drawn as a guide for the eye. Typical random errors from the surface tension measurements are 0.2% except around 50 mN m^–1^ where they amount to 2%.

Surface tension measurements were made on five binary mixtures with C_12_-DTPA and SDS at different molar ratios. The molar ratios are expressed in terms of the mole fraction of C_12_-DTPA, α, on a surfactant only basis. In Figure 5, the surface tensions for C_12_-DTPA, SDS, and the five different mixtures are shown as a function of total surfactant concentration.

Since the purpose of this study was to evaluate the interactions in monolayers at the air/water interface, the analysis of the surface tension plots will focus mainly on the lower concentration range, where no micelles are present in the systems. It is worth noticing from Figure 5 that the unconventional increase in surface tension at higher concentrations that is found for the chelating surfactant, which was discussed in the Introduction, is reduced significantly at high mole fractions of SDS.

In applications it is often found to be beneficial to use mixtures of surfactants rather than single component systems.^22^ The data in Figure 5 have been analyzed with respect to possible synergism in the mixed monolayer by using the concepts of surface tension reduction effectiveness and surface tension reduction efficiency. Surface tension reduction effectiveness is defined by the maximum reduction in surface tension compared to the pure solvent.^9^ Synergism in this respect is found when a mixture of two surfactants shows a minimum surface tension value lower than that of the individual surfactants. This is the case for all of the mixtures shown in Figure 5. Also, note how all mixtures reach the same minimum surface tension value and, thus, show the same surface tension reduction effectiveness. This is most probably correlated to the results presented in Figure 4, where it was shown that the surface composition deviated from the solution composition by tending toward a certain molar ratio between the two surfactants over most of the solution compositions examined. At the concentration examined in Figures 4, 1 mmol L^–1^, the interactions in the surface layer resulted in a surface composition of 0.75 mole fraction of C_12_-DTPA.

**Figure 6:**
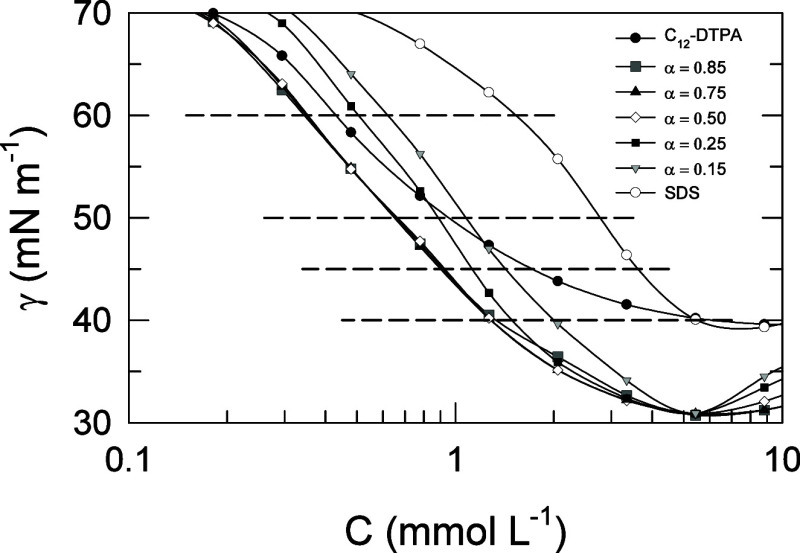
Surface tension as a function of total surfactant concentration for C_12_-DTPA, SDS, and the five mixtures at different mole fractions of C_12_-DTPA (α). The lines between data points are drawn as a guide for the eye. The four surface tension levels chosen for analysis are indicated as horizontal dashed lines at 40, 45, 50, and 60 mN m^–1^, respectively. Typical random errors from the surface tension measurements are 0.2% except around 50 mN m^–1^ where they amount to 2%.

The lower concentration regions are conveniently analyzed by choosing specific values of the surface tension. In this study, four different surface tension levels were chosen: 40, 45, 50, and 60 mN m^–1^. These are shown in Figure 6, which is an enlargement of Figure 5.

Surface tension reduction efficiency is a measure of the surfactant concentration needed to reduce the surface tension to a certain level, compared to the pure solvent; the lower the amount of surfactant needed, the higher is the efficiency.^9^ C_12_-DTPA shows a higher efficiency than SDS, evident from the position of its surface tension plot to the left (toward lower concentrations) compared to that of SDS. This is in line with the results from surface excess shown in Figure 2, for equimolar mixtures of the two surfactants, where C_12_-DTPA was found to be primarily adsorbed to the surface at low total concentration. Synergism in surface tension reduction efficiency is found in the cases when a lower concentration of a mixture of two surfactants, than of the individual surfactants, is needed to reduce the surface tension to a specific level. For the four different surface tension levels chosen, it can be seen in the figure that for the two lowest surface tension levels, all mixtures show synergism since they all lie to the left of the curves for the individual surfactants. At 50 mN m^–1^, on the other hand, the mixture with the lowest mole fraction of C_12_-DTPA does not show synergism, and at 60 mN m^–1^, the two mixtures with the lowest mole fraction of C_12_-DTPA do not show synergism.

### Interaction Parameters in Mixed Monolayers from Surface Tension Measurements—Theoretical Framework

In order to further examine the interactions between the two investigated surfactants, interaction parameters for mixed monolayer formation at the air–water interface (β^σ^) were calculated from the surface tension plots. The superscript σ is used to highlight that the interactions take place in the surface layer. The calculations are based on the surface tension reduction efficiency of the binary mixtures,^9,13,23^ following the framework of interaction parameters for mixed micelle formation using the regular solution theory as described by Holland and Rubingh.^6^ Interactions in surface layers can be evaluated at any arbitrary surface tension level, up to the onset of the formation of micelles. Here, calculations have been made at the four surface tension levels chosen (40, 45, 50, and 60 mN m^–1^, see Figure 6). Obviously, the surface tension decreases with increasing surfactant concentration in the region up to micellization. It is important to realize in such analysis that one surface tension level does not correspond to the same surfactant concentration for all the mixtures but rather to a certain range of surfactant concentrations, since the mixtures, as well as the individual surfactants, possess different surface tension reduction efficiency. Note how this differs from the neutron reflection measurements described in the section The distribution of surfactants in mixed monolayers–effects of surfactant composition that were performed on each mixture at a fixed total surfactant concentration of 1 mmol L^–1^. It is unfortunately not straightforward to design experiments that allow for direct comparison between surface tension and neutron reflection measurements due to the difference in how the results are evaluated.

For the purpose of calculating interaction parameters for mixed monolayers, we need to define a few quantities. The concentration of C_12_-DTPA and SDS, respectively, needed to reduce the surface tension to the specific level are referred to as C_1_^0^ and C_2_^0^. As stated above, four surface tension levels were investigated in this respect. The mole fraction of C_12_-DTPA in the binary mixtures, on a surfactant only basis, is referred to as α. The total surfactant concentration at a specific α needed to reduce the surface tension to the specific level is referred to as *C*_mix_. *C*_1_^0^, *C*_2_^0^, and *C*_mix_ are determined at each surface tension level from the experimental surface tension data, i.e., from the four horizontal lines in Figure 6.

*X*_1_, the mole fraction of surfactant 1 (on the basis of surfactant molecules only) in the mixed monolayer, is calculated from *C*_1_^0^ and *C*_2_^0^ of the individual surfactants and *C*_mix_ of their mixture at α by solving eq 2 numerically^13,23^
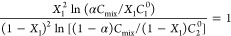
2

*X*_1_ is then substituted into eq 3 to calculate the interaction parameter, β^σ^ for the surfactants in the mixed monolayer at the air/water interface^13,23^
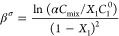
3

The parameter β^σ^ reflects the magnitude of the interactions in the mixed system relative to the self-interactions for the two individual surfactants. The value of the interaction parameter is proportional to the free energy of mixing, and the stronger the attraction of the components in the mixture relative to the self-interactions for the individual surfactants, the more negative the value of β^σ^. Mixtures containing amphoteric surfactants and ionic surfactants are known to show negative β^σ^ values indicative of more favorable interactions between the different surfactants than between the individual components due to the adapted degree of protonation of the amphoteric surfactant.^9−12^ A similar situation occurs in mixtures containing pH-sensitive zwitterionic surfactants that are capable of either accepting or donating a proton to acquire a net negative or positive charge.^10^ Note how this differs from amphoteric surfactants, which are capable of both accepting and donating protons; it should also be noted that pH-sensitive zwitterionic surfactants are often referred to as just zwitterionic in the literature. For example, n-dodecyl-*N*,*N*-dimethylaminoacetate (C_12_-betaine) contains a carboxyl group that can become protonated and, consequently, interacts stronger with anionic surfactants than with cationic surfactants.^21^ However, there are also studies showing strong interactions between simple zwitterionic surfactants and ionic surfactants, and this seems to be particularly pronounced in mixtures with anionic surfactants.^24^ In mixtures of two surfactants with the same charge, interaction parameters close to zero are expected since mixing of the two surfactants will not reduce the entropy penalty of counterion binding. Despite the fact that C_12_-DTPA would have a net negative charge on its own at the examined pH (in contrast to C_12_–betaine that may have a net zero or positive charge), it is clear from the values in Table 2 that there are attractive interactions between C_12_-DTPA and the anionic SDS. This is in line with discussions above regarding increased protonation of C_12_-DTPA when adsorbed at the surface, leading to attractive interactions with SDS through reduced surface charge density and thereby increased entropy of the counterions.

**Table 2:** Calculated Interaction Parameters Between C_12_-DTPA and SDS for Each of the Five Examined Mole Fractions of C_12_-DTPA in Solution (α) and Each of the Four Examined Surface Tension Levels^a^

	β^σ^			
α	60 mN m^–1^	50 mN m^–1^	45 mN m^–1^	40 mN m^–1^
0.85	–3.0	–3.6	–4.4	–6.9
0.75	–2.7	–3.3	–4.1	–6.4
0.5	–2.9	–3.3	–3.9	–6.0
0.25	–2.4	–3.0	–3.7	–5.7
0.15	–2.4	–2.9	–3.5	–5.1

a Typical uncertainties for β values are 5% or less.

As shown in Table 2, there is not one single β^σ^ value that describes the interactions between C_12_-DTPA and SDS in the surface layer for all conditions; rather the interaction parameter depends on both concentration (or surface tension level) and solution composition (α).

The interactions in the system become stronger when the total surfactant concentration increases, as seen from the more negative β-parameter at the lower surface tension levels, i.e., going from left to right in the table. This can be understood by comparing Figure 2 with 6 and considering how the composition of the surface layer changes with the total surfactant concentration. According to Figure 2, C_12_-DTPA dominates the surface layer at the lowest concentration examined (0.5 mmol L^–1^), whereas at a concentration of 3.5 mmol L^–1^, the two surfactants are present in equal amounts. Figure 6 reveals that the highest surface tension level (60 mN m^–1^) corresponds roughly to a concentration of 0.5 mmol L^–1^ or slightly lower, and the lowest surface tension level (40 mN m^–1^) corresponds approximately to a concentration of about 2 mmol L^–1^. This means that the change in concentration from the level of 60 mN m^–1^ to the level of 40 mN m^–1^ in Figure 6 is associated with a composition change in the surface layer, from consisting primarily of C_12_-DTPA to more equal amounts of the two surfactants. A mechanism for this change in composition has already been discussed in the section The distribution of surfactants in mixed monolayers—effects of total surfactant concentration. Here, we note that more negative β values correlate with the surface composition moving toward more similar concentrations of the two surfactants. The interaction also becomes stronger with increasing mole fraction of C_12_-DTPA in the solution (α), going up a column in the table. This effect becomes more pronounced with increasing total surfactant concentration and lower surface tension level. This may also be a consequence of the amphoteric nature of the chelating surfactant.

### Fitted Interaction Parameters by Least-Square Fit

Interaction parameters are often reported in the literature as a single value describing a system over a whole range of solution compositions. To obtain this, one mean value for the interaction parameter at each specific surface tension level was calculated. These mean values were used for further calculations to finally come up with one fitted interaction parameter at each specific surface tension level according to the following procedure. To begin with, values of *C*_mix_ were calculated, as opposed to the above determined *C*_mix_ values obtained directly from the horizontal lines in surface tension plots (Figure 6). The calculated *C*_mix_ values were derived from the mean values for the interaction parameters using eqs 4, 5aa, and 5ab, and with *X*_1_ ranging from 0 to 1 with a fixed increment of 0.1,^23^
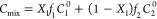
4

*f*_1_ and *f*_2_ are the activity coefficients of surfactants 1 and 2 in the mixed monolayer. When there is no net interaction between the two surfactants, i.e., in the ideal case, *f*_1_ = *f*_2_ = 1. However, in the nonideal case described here, the activity coefficients of the surfactants in the mixed monolayer can be calculated from the regular solution theory:
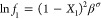
5a
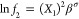
5b

Calculated values of α, as opposed to the above predetermined α values from sample preparation, were then obtained using eq 3 and solving for α, again using the mean values for the interaction parameters and with *X*_1_ ranging from 0 to 1 with a fixed increment of 0.1. Finally, an overall optimized interaction parameter (β^fit^) at each specific surface tension level could be obtained, see Table 3, by an iterative process starting from the mean values of the interaction parameters and minimizing the sum of the square of the difference between the calculated *C*_mix_ values and the *C*_mix_ values obtained directly from the experimental surface tension data.

**Table 3:** Fitted Interaction Parameters Between C_12_-DTPA and SDS for Each of the Four Surface Tension Levels^a^

surface tension level (mN m^–1^)	β^fit^
60	–2.5
50	–3.1
45	–3.8
40	–6.0

a Typical uncertainties for values of β are 5% or less.

**Figure 7:**
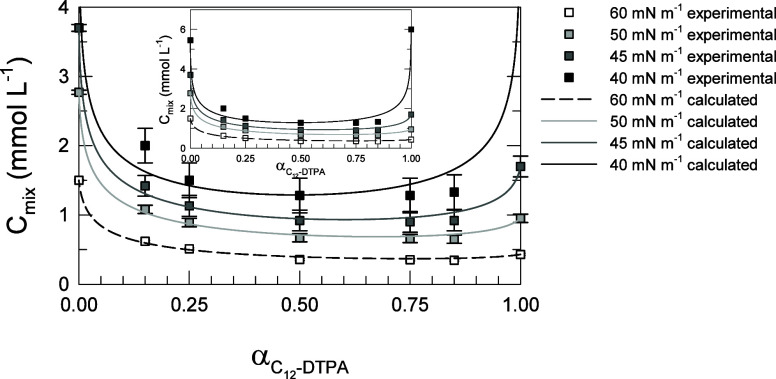
C_12_-DTPA and SDS mixtures. *C*_mix_ as a function of α (mole fraction C_12_-DTPA in solution) for the four selected surface tension levels. C_1_^0^ (C_12_-DTPA), C_2_^0^ (SDS), and *C*_mix_ values derived from the surface tension data from Figure 6 at the different α are shown as squares, and the calculated *C*_mix_ values as a function of calculated α, based on the fitted interaction parameters, are shown as lines. The figure shows an enlargement, and the full graph with all data is shown as an insert.

Calculated *C*_mix_ values as a function of calculated α, based on the fitted interaction parameters, are shown in Figure 7. The C^0^ and *C*_mix_ values derived from the surface tension data from Figure 6 are also indicated in the figure (as squares). The C^0^ values at the four surface tension levels are listed in Table 4 in the next section. When the C^0^ of the two surfactants are similar, as seen at the surface tension level of 40 mN m^–1^, the curve of *C*_mix_ as a function of calculated α is symmetrical. When the difference between C_1_^0^ (C_12_-DTPA) and C_2_^0^ (SDS) becomes larger, the curves become asymmetrical.

**Table 4:** Concentration of C_12_-DTPA (C_1_^0^) and SDS (C_2_^0^) Needed to Reduce the Surface Tension to the Specific Level, C_1_^0^/C_2_^0^, and Optimum Composition for the Four Surface Tension Levels

surface tension level (mN m^–1^)	C_1_^0^ (C_12_-DTPA) (mmol L^–1^)	C_2_^0^ (SDS) (mmol L^–1^)	C_1_^0^/ C_2_^0^	optimum composition
60	0.43 ± 0.02	1.5 ± 0.02	0.23	0.75
50	0.95 ± 0.07	2.8 ± 0.02	0.34	0.67
45	1.7 ± 0.2	3.7 ± 0.02	0.46	0.60
40	6.0 ± 0.2	5.5 ± 0.02	1.1	0.49

Since the calculated *C*_mix_ values are based on one overall fitted interaction parameter for each surface tension level, and the values determined directly from surface tension data resulted in varying interaction parameters as seen in Table 2, there is a certain discrepancy between calculated and experimentally derived values. Obviously, the discrepancy is larger at the lower surface tension levels, where the largest variation in the interaction parameter was found (Table 2).

### Maximum Synergism in the Surface Tension Reduction Efficiency—The Optimum Composition

**Figure 8:**
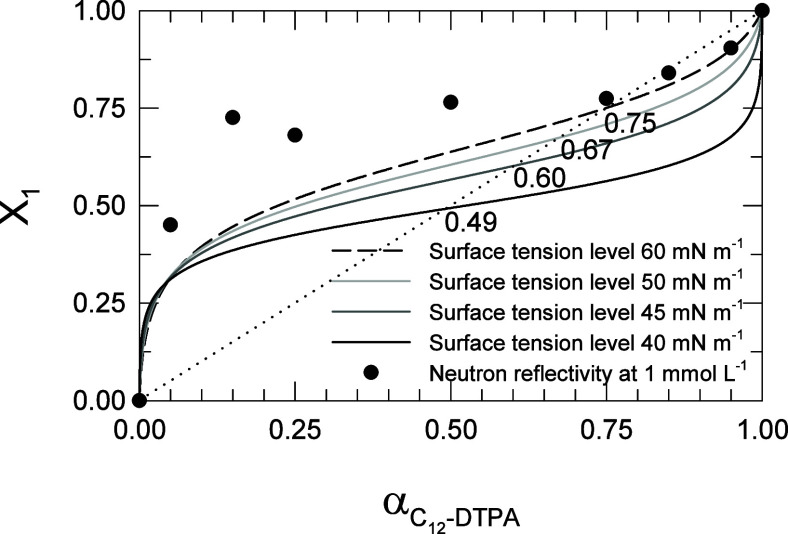
C_12_-DTPA and SDS mixtures. Mole fraction C_12_-DTPA at the surface, *X*_1_, as a function of mole fraction C_12_-DTPA in solution, α. The lines represent *X*_1_ ranging from 0 to 1 (at a fixed increment of 0,1) as a function of calculated α at each of the four selected surface tension levels. The values of the optimum composition are indicated in the figure, where each curve crosses the diagonal. The dots represent the experimentally determined *X*_1_ from neutron reflection measurements at a total surfactant concentration of 1 mmol L^–1^.

The concept of surface tension reduction efficiency was discussed in the section Surface tension measurements and synergism in mixed monolayers. The less surfactant that is needed to reduce the surface tension to a certain level, the higher is the surface tension reduction efficiency. Synergism is found when a lower concentration of a mixture of two surfactants than of the individual surfactants is required to reduce the surface tension to a specific level. In other words, a minimum in the *C*_mix_ value, plotted as a function of mole fraction of C_12_-DTPA in solution (α) (Figure 7) indicates a maximum synergism in the surface tension reduction efficiency. This maximum synergism appears when the solution composition matches the composition in the mixed monolayer, i.e., when the mole fraction of C_12_-DTPA in solution equals the mole fraction of C_12_-DTPA at the surface. This point is described in the literature as the optimum composition.^9^ Using the fitted interaction parameters, the mole fraction of C_12_-DTPA at the surface, *X*_1_, ranging from 0 to 1 with a fixed increment of 0.1, was plotted in Figure 8 as a function of calculated α, the mole fraction of C_12_-DTPA in the solution, to obtain the optimum compositions for each of the four selected surface tension levels. The experimentally determined *X*_1_ values from neutron reflection measurements at a total surfactant concentration of 1 mmol L^–1^ (from Figure 4b) are also included in the figure.

The optimum composition, and thus the maximum synergism in surface tension reduction efficiency, is found where the curve for a given surface tension level crosses the diagonal, i.e., the dotted line in Figure 8. The values for optimum composition are indicated in Figure 8 and are listed in Table 4 together with C^0^ for the two surfactants.

As discussed in the section The distribution of surfactants in mixed monolayers—Effects of surfactant composition, the optimum composition changes with total surfactant concentration, but nonetheless, the strive for keeping the surface composition close to the specific optimum over a large span of solution compositions persists.

When the C^0^ of two surfactants in a binary mixture are similar, the optimum composition is close to 0.5, but if the C^0^ of one of the surfactants is lower, the optimum composition is shifted toward increased mole fraction of that surfactant.^9^ Starting from the highest surface tension level examined (60 mN m^–1^) where C_12_-DTPA has a significantly lower C^0^ than SDS (0.43 compared to 1.5 mmol L^–1^), it can be concluded that C_12_-DTPA dominates the surface as seen from that curve crossing the diagonal at an optimum composition of 0.75 mole fractions of C_12_-DTPA. As the surface tension level decreases (and the concentration increases), the optimum composition gradually shifts toward increased amounts of SDS as the C^0^ of the two surfactants become more comparable. Finally, a value close to 0.5 is reached at the lowest surface tension level of 40 mN m^–1^, where the C^0^ values of the two surfactants are close to each other. This is consistent with the results from neutron reflectivity discussed in the section The distribution of surfactants in mixed monolayers—Effects of total surfactant concentration and shown in Figure 2a. The chelating surfactant was found to dominate the surface excess at low total surfactant concentrations, and increasing the concentration caused SDS to be enriched in the surface layer at the expense of C_12_-DTPA resulting in equimolar concentrations of the two surfactants at 3.5 mmol L^–1^.

The stronger the interactions in the mixed monolayer, the higher is the tendency for the system to tend toward the optimum composition in the surface layer over a large span of solution composition. In other words, the slope of the middle part of the curve in Figure 8 reflects the strength of the interactions in the mixed monolayer, with strong interactions leading to a flat middle part of the curve. In accordance with the interaction parameter having the largest negative value at the lowest surface tension level (40 mN m^–1^, see Table 3), that curve is the one that exhibits the flattest central part, rendering the composition in the surface layer close to 0.5 over a large range of solution compositions.

### Correlation Between Mole Fraction of C_12_-DTPA in the Surface Layer from Neutron Reflection and from Surface Tension Measurements

**Figure 9:**
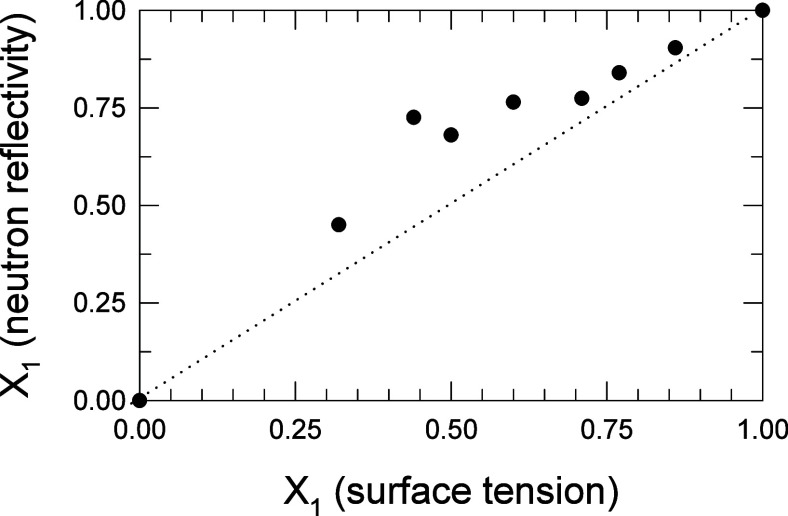
C_12_-DTPA and SDS mixtures. The correlation between the mole fraction of C_12_-DTPA at the surface (*X*_1_) from neutron reflection and from surface tension measurements. The surface tension level of 50 mN m^–1^ was chosen for this comparison. Typical uncertainties are 3%.

To further compare the results from the two techniques with respect to the amount of C_12_-DTPA in the surface layer, *X*_1_ derived from neutron reflection measurements are plotted as a function of *X*_1_ derived from surface tension measurements, for the respective α, see Figure 9.

One should remember, as pointed out already, that there is no straightforward way of designing neutron reflection experiments that allows for an exact comparison at a certain concentration with results for a given surface tension. For surface tension measurements, calculated values of α (based on fitted β^σ^, using eq 3 and solving for α) were used, and the corresponding *X*_1_ values were plotted in the figure. The surface tension level of 50 mN m^–1^ was chosen for this comparison since that was the level that best matched the 1 mmol L^–1^ samples in the neutron reflection measurements. Since calculated values of mole fraction from surface tension measurements are based on a fitted interaction parameter, and it was shown in Table 2 that there is not one single β^σ^ value that describes the interactions under all conditions examined, a certain discrepancy from the mole fraction derived from neutron reflection measurements can be expected. Although the mole fraction of C_12_-DTPA in the surface layer derived from neutron reflection measurements is systematically higher than the mole fraction derived from surface tension measurements, there seems to be reasonably good agreement between the two data sets.

## Conclusions

The combined use of neutron reflection measurements and determination of surface tension provides specific insights into the interaction of molecules in a surface layer. In particular, the direct observation of the composition allows models based on thermodynamic theories to be examined.

The results presented for the pure anionic surfactant, SDS, both as regards surface tension as a function of concentration and surface excess at the air/water interface for a 1 mmol L^–1^ solution are in good agreement with those reported by Xu et al.,^25^ where measurements on carefully purified samples are compared with other values in the literature. The data for the chelating surfactant alone correspond with those reported in our previous work.^2,3^

In accordance with the hypothesis put forward for this study, the amphoteric nature of the chelating surfactant greatly influences its surface activity, the interactions with another surfactant, and consequently also the composition of mixed surface layers. The composition of the surface layer of a mixture containing an amphoteric surfactant may change dramatically with concentration since this involves a change in the ionic strength and subsequently a change in the balance between competing entropic influences in the system. A considerable consequence of the amphoteric structure is the strong favorable interactions between the surfactants in the mixed surface layers. It is of particular note that this occurs even though it is expected that both of the individual surfactants would be negatively charged at the studied solution pH of 5. The interaction parameter between surfactants in a surface layer is not constant with the concentration or surface packing. These observations are apparently consequences of the facility with which the chelating surfactant can adapt its conformation and ionization. The contrasting behavior of C_12_-DTPA to that of simpler zwitterionic surfactants such as n-dodecyl-*N,N*-dimethylaminoacetate (C_12_-betaine) and *N*-dodecyl-*N,N*-dimethyl-3-ammonio-1-propane sulfate (C_12_-sulphobetaine) is seen in the comparison with the results of Hines et al, Li et al., and Wydro and Paluch.^20,24^,^26^

Linearity of correlation between the mole fraction of C_12_-DTPA in the surface layer from neutron reflection and from surface tension measurements shows that the simple thermodynamic mixing theory is a reasonable approximation provided appropriate surface tension values are chosen.

This study provides novel insights into the behavior of mixed surfactant systems containing amphoteric surfactants and may have important implications for various fields, including material science and industrial applications.

## Supporting Information Available

Plots of neutron reflectivity data with model for fitted monolayer and tables of fitted parameters (PDF)
