# Supplementary material for: Impact of the Amphoteric Nature of a Chelating Surfactant on its Interaction with an Anionic Surfactant: A Surface Tension and Neutron Reflectivity Study of Binary Mixed Solutions
Source: ACS Omega. 2024 Feb 26;9(10):11366–76. doi: 10.1021/acsomega.3c07547 (PMC10938333; doi:10.1021/acsomega.3c07547)
Supplement: Supplementary file 1 — ao3c07547_si_001.pdf [file ao3c07547_si_001.pdf]

Supporting Information

Impact of the Amphoteric Nature of a Chelating surfactant on Its Interaction with an Anionic Surfactant: A Surface Tension and Neutron Reflectivity Study of Binary Mixed Solutions

Ida Svanedal[*,a] , Håkan Edlund[*,a] , Magnus Norgren[a] , Sushil K. Satija[b] , Adrian R. Rennie[c]

[a]Surface and Colloid Engineering, FSCN Research Centre, Mid Sweden University, SE-851 70 Sundsvall, Sweden.

[b]NIST Center for Neutron Research, 100 Bureau Drive, MS 6100, Gaithersburg, MD 20899-6100, USA.

[c]Macromolecular Chemistry and Centre for Neutron Scattering, Uppsala University, Ångström Laboratory, Box 520, SE-75121 Uppsala, Sweden.

*Corresponding authors: Ida Svanedal, e-mail ida.svanedal@miun.se, telephone +46(0)10-1428559; Håkan Edlund, e-mail hakan.edlund@miun.se, telephone +46(0)70-5251519

Plots of neutron reflectivity data with model for fitted monolayer and tables of fitted parameters.

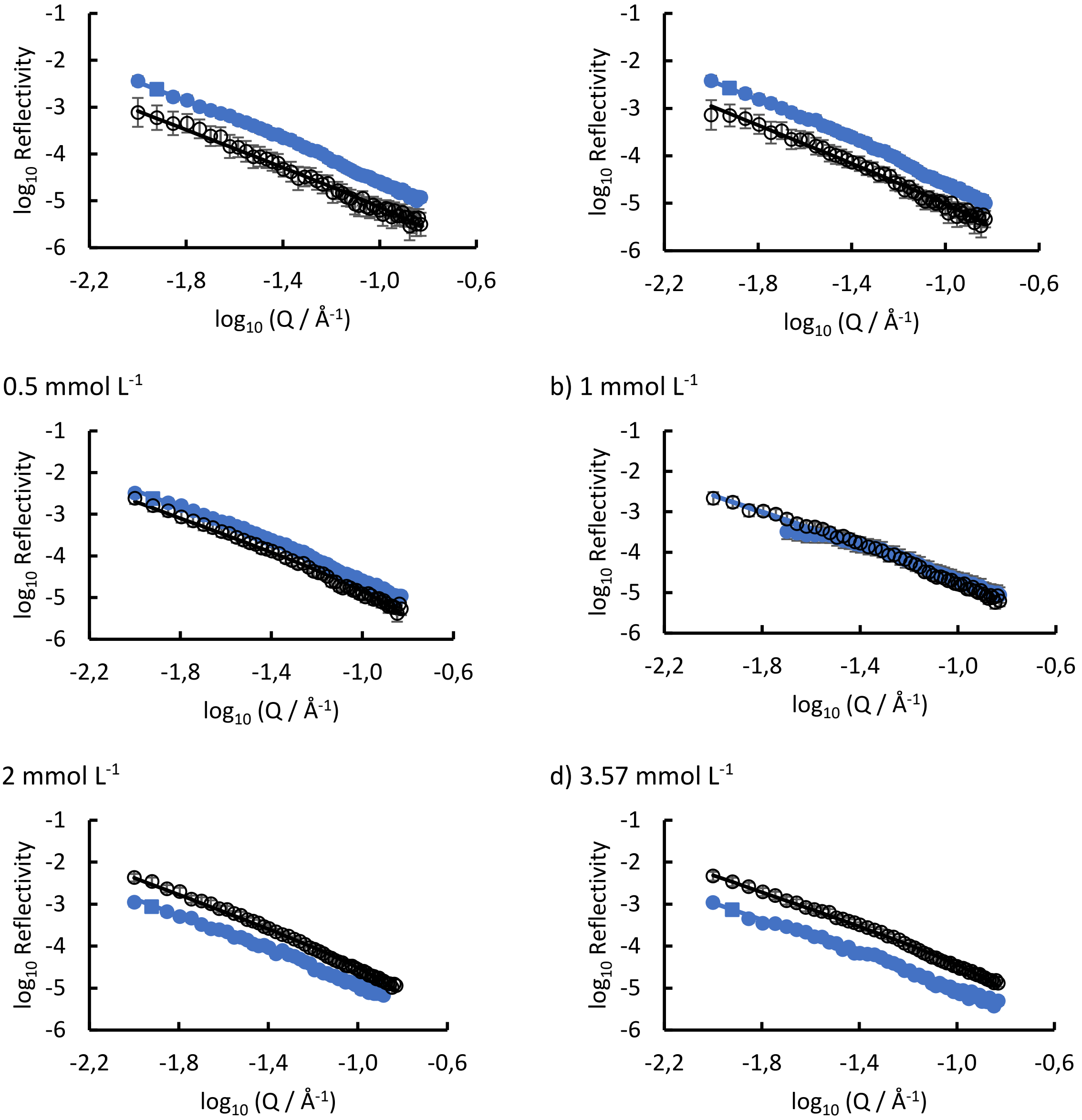

a) 0.5 mmol L^{-1}

b) 1 mmol L^{-1}

c) 2 mmol L^{-1}

d) 3.57 mmol L^{-1}

e) 10 mmol L^{-1}

f) 20 mmol L^{-1}

Figure S1. Neutron reflectivity data at different total concentrations of surfactant for the equimolar composition ratio of deuterated C_{12}-DTPA / hydrogenous SDS (■) and hydrogenous C_{12}-DTPA / deuterated SDS (○) with the corresponding combined fits, shown as continuous lines, for mixed monolayers of surfactant as described in the text. The parameters for the reflectivity models are shown in Table S1.

Table S1. Parameters derived from fitting a mixed monolayer model to the neutron reflectivity data for equimolar mixtures of C_{12}DTPA and SDS.

C / mmol L^{-1}	n_1 / Å^{-2}	Δn_1 / Å^{-2}	n_2 / Å^{-2}	Δn_2 / Å^{-2}	t_1 / Å	Δt_1 / Å	t_2 / Å	Δt_2 / Å
0.5	0.0119	0.0002	0.0028	0.0001	16.4	1.0	11.9	1.1
1	0.0125	0.0002	0.0038	0.0002	18.5	0.9	13.2	1.3
2	0.0122	0.0002	0.0073	0.0002	18.9	0.8	20.2	0.7
3.57	0.0098	0.0001	0.0099	0.0002	13.5	0.8	19.4	0.5
10	0.0069	0.0001	0.0156	0.0004	12.8	1.3	19.9	0.7
20	0.0060	0.0001	0.0173	0.0003	11.5	1.3	18.8	0.7

The surface excess, n, for each surfactant is given as the number of molecules per square Ångström. The quantities, t_1 and t_2, represent the layer thickness when respectively the C_{12}DTPA and SDS are deuterated in the mixture that was measured. The uncertainties (Δ) represent two standard deviations as derived from the least squares model fitting. As the background signal was subtracted in the data reduction procedure, the parameter for background was constrained to zero in the fit procedure. The conversion to surface excess of each component, Γ, in units of moles per unit area is simply made by dividing n by Avogadro's constant and making the appropriate conversion to the chosen units of area. The total excess is $n_1 + n_2$ and the composition in the surface X_1 is $n_1 / (n_1 + n_2)$ and X_2 is $n_2 / (n_1 + n_2)$.

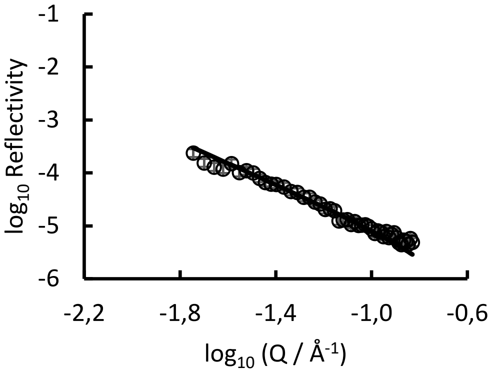

a) α = 0.0

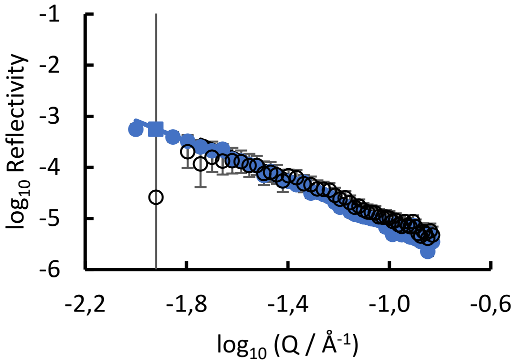

b) α = 0.05

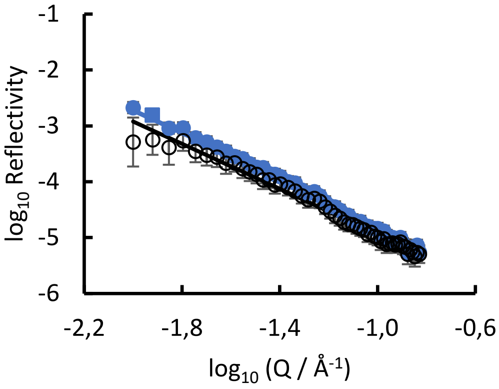

c) α = 0.15

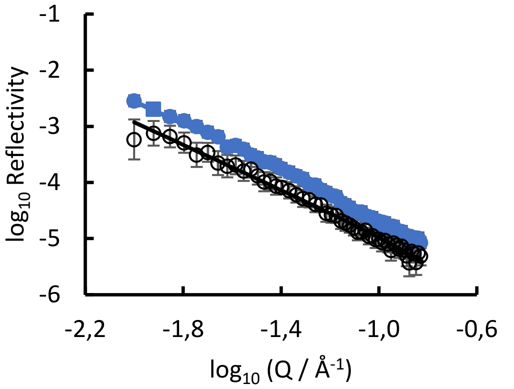

d) α = 0.25

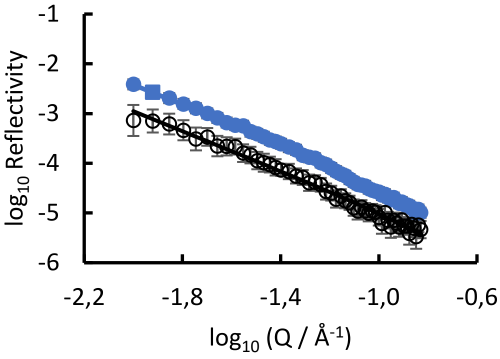

e) α = 0.50

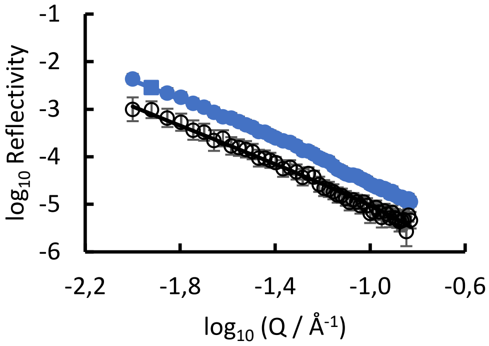

f) α = 0.75

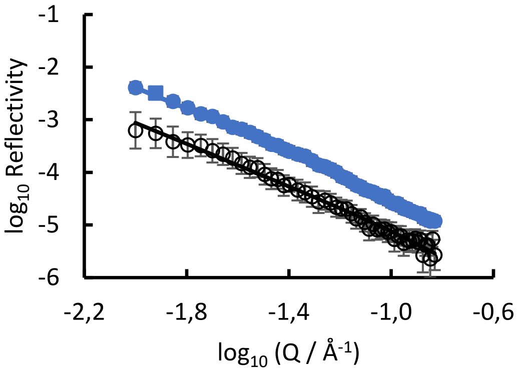

g) α = 0.85

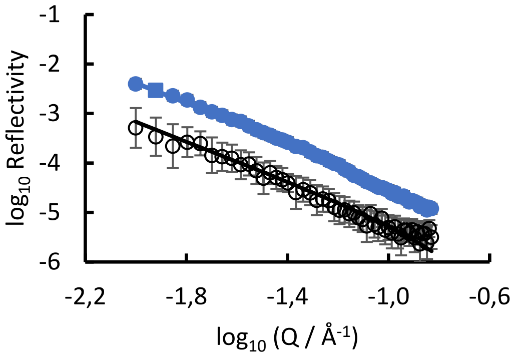

h) α = 0.95

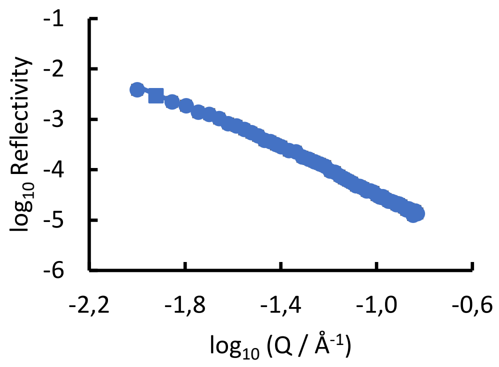

i) α = 1.0

Figure S2. Neutron reflectivity data at different composition ratios of deuterated C_{12}-DTPA / hydrogenous SDS (■) and hydrogenous C_{12}-DTPA / deuterated SDS (○) with the corresponding combined fits, shown as continuous lines, for mixed monolayers of surfactant as described in the text. The total surfactant concentration is 1 mmol L^{-1}. The parameters for the reflectivity models are shown in Table S2. Figure S2 e) is the same as Figure S1 b) and is reproduced here to provide the complete series of compositions studied.

Table S2. Parameters derived from fitting a mixed monolayer model to the neutron reflectivity data for different mixtures of C_{12}DTPA and SDS at a total surfactant concentration of 1 mmol L^{-1}.

α	n_1 / Å^{-2}	Δn_1 / Å^{-2}	n_2 / Å^{-2}	Δn_2 / Å^{-2}	t_1 / Å	Δt_1 / Å	t_2 / Å	Δt_2 / Å
0.00	0	0	0.0090	0.0002	0	0	15.0	0
0.05	0.0056	0.0001	0.0072	0.0002	10.1	2.9	17.0	0
0.15	0.0087	0.0002	0.0059	0.0001	17.9	2.2	9.7	1.1
0.25	0.0106	0.0001	0.0050	0.0005	16.8	0.8	12.5	0.9
0.50	0.0125	0.0002	0.0038	0.0002	18.5	0.9	13.2	1.3
0.75	0.0129	0.0002	0.0038	0.0002	18.1	0.9	15.2	1.3
0.85	0.0130	0.0002	0.0025	0.0002	18.3	0.9	13.2	1.3
0.95	0.0131	0.0002	0.0007	0.0002	18.6	1.2	18.0	0.0
1.00	0.0138	0.0001	0	0	17.2	0.6	0	0

The quantity α is the mole fraction of C_{12}-DTPA in the solution. Other parameters are as described in the note to Table S2.
